# Partial Necrosis Consequence of the Infection Spreading from an Adjacent Apical Periodontitis: A Case Report

**DOI:** 10.22037/iej.v13i3.22089

**Published:** 2018

**Authors:** Saeed Asgary, Leyla Roghanizadeh

**Affiliations:** a *Iranian Center for Endodontic Research, Research Institute of Dental Sciences, Shahid Beheshti University of Medical Sciences, Tehran, Iran; *; b *Dental Research Center, Research Institute of Dental Sciences, Dental School, Shahid Beheshti University of Medical Sciences, Tehran, Iran*

**Keywords:** Anachoresis, Apical Periodontitis, Calcium-Enriched Mixture, CEM Cement, Dental Pulp Necrosis, Endodontic, Spread of Infection

## Abstract

As the dental pulp could not be directly inspected before endodontic treatment, indirect evaluation of the pulp status *via* (para)/clinical tests should be performed which need careful inspection. This report presents a root-treated right maxillary first molar with recurrent abscess formation and a radiolucent periradicular lesion surrounding the distobuccal root of the right maxillary second molar. The patient underwent surgical retreatment, employing CEM root-end filling, which resulted in no relief from sign/symptoms. In the cone-beam computed tomography (CBCT), the relationship of the lesion with the mesio-buccal root of the second maxillary molar was detected. Despite the latest tooth showed positive responses to pulp sensibility tests, endodontic therapy was planned for it. During treatment, it became clear that the mesiobuccal canal pulp was necrotic, although vital pulp tissues were present in two other root canals. Following treatment, full recovery from all discomforts was obtained and the lesion healed after 18 months. This case showed that a more complicated evaluation such as CBCT should be used for diagnosis of perpetuated lesions. Furthermore, it might be probable that root canals of vital teeth become necrotic due to involvement in the adjacent apical lesion, a phenomenon known as anachoresis.

## Introduction

Apical periodontitis is the sequel of the dynamic encounter between an endodontic infection and the host’s defense, which results in inflammation and destruction of periradicular tissues [1]. If apical periodontitis remains after root canal treatment, it is considered as a persistent lesion, usually attributed to failure of endodontic treatment [2]. Investigations show such lesions could be the result of persistent primary or secondary intraradicular, or extraradicular infections with species resisting and proliferating in burdensome environments; some inflammatory or immunologic reactions may be responsible, as well [3, 4]. 

For correct diagnosis and verification the origin of periradicular lesions, assessment of the health status of the pulp in involved teeth is necessary [5]. Pulp sensibility tests (thermal and electrical) have limitations, and false responses can occur. Vitality tests, which are able to directly assess the blood flow within the dental pulp, are better indicators of the pulp health status; however, sensibility tests still have satisfactory validity and accuracy values to indirectly determining the state of pulpal health in common clinical practice [6-8].

Radiographic evaluations play a crucial role in diagnosis of periradicular lesions. The extent of the lesion, number of roots and root canals, detection of the involved root or roots, and whether a lesion around one root has a communication pathway to another root, should be investigated through the radiographic examinations [9]. The limitation of periapical radiographs in rendering information in two dimensions lead clinicians to use computed tomography (CT) scans [10].

This report presents a case of apical periodontitis assumed to be related to a failed conventional root canal therapy of a maxillary first molar, which perpetuated even after surgical retreatment. Finally, it was proved to be attributed to the adjacent partially-vital maxillary second molar, which had given normal positive responses in pulp sensibility tests.

**Figure 1:**
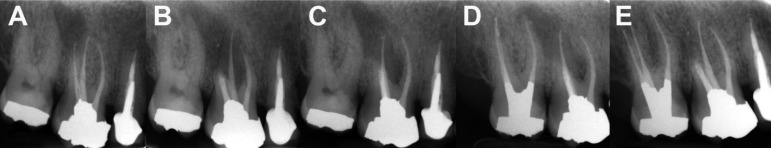
Periapical images: *A)* Preoperative; *B)* Postoperative image after distal root-end surgery of right maxillary first molar; *C)* Postoperative two-month follow-up; *D)* Post-RCT image of the maxillary second molar; *E)* 18-month follow-up: healed periradicular lesion

**Figure 2:**
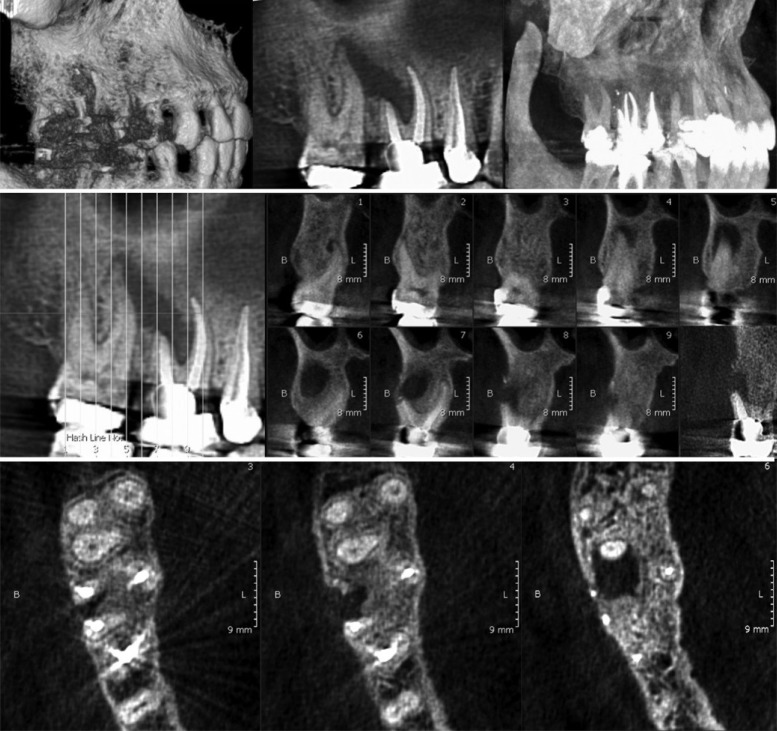
CBCT views showing obvious relationship between the mesiobuccal root of the maxillary second molar and the lesion

## Case Report

A 51-year-old male with no history of systemic disease was admitted to the endodontic department of a private dental clinic. He was suffering from recurrent abscess formation in the right maxillary buccal vestibule near the right upper first molar. Radiographic examination (Figure 1A) revealed a circumscribed periapical lesion contiguous to the distobuccal root of the right upper first molar (tooth #16), extended to the mesial of mesiobuccal root of the adjacent second molar (tooth #17). Tooth #16 was root treated and restored with amalgam. The tooth #17, which had an amalgam restoration, had positive responses to an electrical pulp tester (Parkell, Edgewood, NY, USA), and cold test with Endo-Frost (Coltène-Whaledent, Langenau, Germany). In the clinical examination, no sinus tract was found. The patient had some tenderness to palpation of the associated buccal gingiva and expressed pain on percussion on tooth #16. Probing depths of gingival sulcus in both teeth were normal (<3 mm). Following all examinations, the lesion presumed to be a symptomatic apical periodontitis ascribed to treatment failure of the distobuccal root; and a surgical endodontic retreatment planned to be done for this root. The patient was informed about the treatment plan and his consent for the operation was obtained.

After a 0.12% chlorhexidine mouth rinse, under local anesthesia with 2% lidocaine plus 1:80000 epinephrine (Darupakhsh, Tehran, Iran), a full mucoperiosteal flap was retracted. The lesion was curetted and the specimen was sent for histopathological examination. Root-end resection, root-end preparation, and root-end filling with calcium-enriched mixture (CEM) cement (BioniqueDent, Tehran, Iran) was performed and the flap was repositioned (Figure 1B). Histopathological evaluation confirmed a granulomatous inflammatory lesion. Although the surgery was satisfactory, there was no amelioration in symptoms and signs in the two-month follow-up (Figure 1C). As there was a doubt about existing a vertical root fracture (VRF) in the involved tooth, taking a cone-beam computed tomography (CBCT) from that region was prescribed. The scan (Figure 2) was inspected carefully and no evidence of VRF was found. Watching all the slices of the scan (Figure 2), the spatial relationship between the lesion and the mesiobuccal root of the adjacent upper right second molar was observed. Consequently, endodontic therapy for maxillary second molar was selected as the treatment plan; however, this tooth exhibited positive responses to pulp sensibility tests. At every step, the patient was informed about the diagnosis procedure. Following his consent, the root canal therapy was planned.

After preparing the access cavity under isolation, it was observed that the mesial root canal had no bleeding/vital pulp. It was necrotic despite the existence of vital pulp in the two other root canals. Cleaning and shaping using full-strength sodium hypochlorite was performed; in the same session, root canals were obturated with gutta-percha (Ariadent, Tehran, Iran) using Roth 808 endodontic sealer (Roth Drug Co., Chicago, IL, USA). Subsequently, the coronal restoration with amalgam was carried out (Figure 1D). One week later, the symptoms and clinical signs were comforted. Complete healing of the periapical lesion in the radiographic examinations was achieved at the 18-month follow-up, proving the correctness of the latest treatment plan (Figure 1E)

## Discussion

In this report, a persistent apical periodontitis is presented, attributing to a root-treated right upper first molar and to its adjacent second molar. The ultimate goal in an endodontic diagnostic procedure would be estimation of the histological status of the pulp and periradicular tissues through the radiographic and clinical data collected [5]. Considering the likelihood of the partial necrosis condition in multi-rooted teeth could help to achieve the correct diagnosis much faster in cases like the reported one.

As the clinicians cannot inspect pulp tissue directly before starting endodontic treatment, indirect evaluation of the pulp status via clinical tests is necessary [8]. The validity of pulp sensibility tests is not 100% [11-13]. While evaluating the viability of nerve fibers and not the health or integrity of the pulpal tissue itself as a measure of its vitality, there might be some false-negative or false-positive responses; which may deflect the true diagnosis in partially necrotic teeth [12, 14]. In case of multi-rooted teeth, the interpretation of the results of the pulp sensibility tests is more complicated. This is because of the probability of necrosis in each of the root canals, while at least one root canal may have vital pulp tissue that has not completely undergone necrosis yet, and has remained responsive to tests [11, 15]. According to the findings of a survey, false-positive responses to a cold test were merely observed in teeth with multiple roots, which was considered to indicate the presence of remaining vital tissue in at least one canal [15]. Another study showed an increased probability of partial necrosis in multi-rooted teeth with a raised volume of pulpal tissue, exhibiting false-positive results in pulp sensibility tests [11].

The spatial relationship between the lesion and the mesiobuccal root of the second molar which assumed to be vital was observed in the CBCT. In some anatomical regions, such as posterior maxilla, interpretation of two-dimensional periapical radiographs would be more difficult. Existence of various anatomical structures, such as maxillary sinus and zygomatic buttress, proximity of maxillary molars’ roots and their overlaps contribute to those difficulties [10]. CBCT scans overcome such problems. Precise root morphology, unidentified root canals, exact location and true extent of lesions and their spatial relationship to anatomical structures, and the actual root to which the lesion is associated can be shown much more evidently in the scans [16]. According to the results of an investigation, CBCT scans can help decision making in endodontic retreatments, especially when an apical surgery is needed. Furthermore, infection spreading that originates from maxillary teeth could be obviously recorded by CT scans [17]. Despite those advantages, a CT scan is still expensive and expose the patient a higher radiation dosage than PA radiography [10].

Another point is how the right upper second molar became necrotic, even though no caries, no defect in the restoration, no periodontal disease, and no physical trauma had occurred recently. There are rare cases in the reviewed literature that the periapical infectious lesion of a tooth spread to the root canals of the adjacent teeth and devitalized them. Extension of an apical periodontitis, associated to a mandibular central incisor, to the roots of contiguous teeth has been reported, which the contiguous teeth underwent necrosis. The process was attributed to the proliferation and retrograde entrance of microorganisms from the spreading infectious lesion to the unfilled root canals of the adjacent teeth [18]. Another clinical report described an intact mandibular second premolar becoming non-vital after nonsurgical retreatment of its posterior first molar with a periapical lesion. The bacteria, their by-products, and biological mediators of inflammation were considered the probable etiologic factors for necrosis of the premolar tooth [19]. Such cases confirm the rare possibility of reciprocal infection spread between an apical periodontitis and the adjacent sound teeth. 

Nowadays several calcium silicate based cements have been introduced in dentistry with the aim of different worthy applications in clinical practices like pulp capping, root-end filling, reparative material for perforations and periodontal defects. They can be set in contact with body fluids particularly blood, and can endure dislodging forces while positioning or masticating [20]. Mineral trioxide aggregates (MTA) as a biocompatible biomaterial has excellent sealing ability and satisfactory antimicrobial effects. However, it has some drawbacks in clinical usage such as poor handling characteristics [21]. The bioactive material which has been used in this case is CEM cement. CEM cement has more flow, less film thickness and shorter setting time which may be attributed to the particle size of this biomaterial, causing some superior handling characteristics over MTA [22]. Sealing ability of CEM cement is comparable to MTA, in spite of being higher in CEM. It has been applied in various reparative and regenerative procedures in dentistry such as direct pulp capping of primary teeth, root-end filling, vital pulp therapy, apical plug technique and revascularization procedure in permanent teeth, achieving satisfactory cost-effective clinical results. [23-25]. 

## Conclusion

When a periradicular lesion is persistent in spite of good quality endodontic treatment, more complicated/expensive and less available para-clinical tests _such as cone-beam computed tomography_ should be considered.

There might be a rare possibility of retrograde passage of bacteria from an apical infectious lesion to the adjacent teeth.
